# Neglected Parasitic Infections and Poverty in the United States

**DOI:** 10.1371/journal.pntd.0003012

**Published:** 2014-09-04

**Authors:** Peter J. Hotez

**Affiliations:** 1 National School of Tropical Medicine at Baylor College of Medicine, Houston, Texas, United States of America; 2 Sabin Vaccine Institute and Texas Children's Hospital Center for Vaccine Development, Houston, Texas, United States of America; 3 James A. Baker III Institute for Public Policy at Rice University, Houston, Texas, United States of America; 4 Department of Biology, Baylor University, Waco, Texas, United States of America; Yale School of Public Health, United States of America

The neglected tropical diseases (NTDs) are a group of chronic and disabling infections that occur primarily in settings of extreme poverty and affect over 1,000,000,000 people globally [1]. A selected group of neglected parasitic infections, including some which overlap with the World Health Organization's new list of recognized NTDs [2], are also common in the United States, where they disproportionately affect the poor. The major neglected parasitic infections in the US include Chagas disease, cysticercosis, toxocariasis, toxoplasmosis, and trichomoniasis. These five parasitic infections are considered “neglected” based on their high prevalence, chronic and disabling features, and their strong links with poverty [1], [2]. In contrast, the major intestinal parasitic infections found in the US—cryptosporidiosis, cyclosporiasis, and giardiasis—are mostly acute diarrheal illnesses without significant links to poverty or neglected populations. This review highlights new information (mostly from the last five years) on the major neglected parasitic infections affecting impoverished Americans, with respect to their distribution and unique clinical presentations as well as their surprising links to cardiovascular, respiratory, and neuropsychiatric conditions ordinarily thought of as noncommunicable diseases. Key diagnostic and therapeutic challenges and urgent needs for active surveillance and prevention are also presented.

## Determinants of Neglected Parasitic Infections in the US

NTDs have been shown to flourish in settings of warm climate and extreme poverty found in global subtropical regions similar to the southern US. Indeed, new information suggests that many of the world's NTDs occur predominantly among the extreme poor living in the group of 20 (G20) countries, mostly in in the subtropics, including Brazil, Indonesia, India, China, Saudi Arabia, and Mexico [3]. It is now recognized that several neglected parasitic infections are also widespread in the southern US [4]. This finding is consistent with new US census data indicating that 20 million Americans now live in “extreme poverty” [5], with 1.65 million households (with 3.55 million children) living on less than US$2 per day in a given month [6]—a standard benchmark for global poverty. Today, the states with the highest poverty rates are all in the southern part of the country (Table 1) [7], and the nation's poorest large metropolitan area (McAllen-Edinburg-Mission, Texas) and the eight most impoverished smaller metropolitan areas are located in this region [8]. While there are noncash safety-net programs, including food stamps and public health insurance, that blunt some of the hardship of those living in extreme poverty, there has still been a clear increase in the number of Americans living in poverty over the last 40 years [5]. The underlying basis for why poverty promotes neglected parasitic infections in the southern US is unknown, although factors such as poor housing and sanitation and environmental contamination are likely contributors [5], while so far the links to ethnicity appear to be mainly socioeconomic. Through their chronic and disabling effects on worker productivity, child development, and maternal health [9], it is plausible that neglected parasitic infections could also help perpetuate generational poverty among people of color in the US.

**Table 1:** Geographic distribution of poverty in the United States.

State	Poverty Rate	Rank among US States
Mississippi	21.0	1
New Mexico	19.9	2
Arizona	19.1	3
District of Columbia	19.1	3
Louisiana	18.9	5
Georgia	18.5	6
Texas	17.7	7

Percentage of people in poverty by state using 3-year averages: 2009–11. http://www.census.gov/hhes/www/poverty/data/incpovhlth/2011/tables.html.

Initial efforts to assess the prevalence or incidence of the neglected parasitic infections were hampered by a dearth of available data on these conditions [5], [10]. In response, legislation known as the “Neglected Infections of Impoverished Americans Act” (HR 528) was drafted to raise awareness of these diseases among the general public and subsequently introduced as a bill in 2010 and 2011 [11]. While efforts have since stalled in the US Congress, over the last five years additional information about the neglected parasitic infections has accumulated so that it is possible to begin making more informed statements on their status with regards to prevalence, geographic distribution, and novel associations with illnesses that resemble noncommunicable diseases (Table 2) and on the initial steps required for prevention.

**Table 2:** Neglected parasitic infections in the United States.

Neglected Parasitic Infection	Selected Prevalence Data	Major Risk Factors	Clinical Sequelae in Adults and Children 1	Clinical Sequelae in Adults and Children 2	Congenital Clinical Sequelae	References
Toxocariasis	>21% Seroprevalence among African- Americans (up to 2.8 million African-Americans); >17% seroprevalence in the American South	African-American race, male sex, poverty, low education level, lead ingestion, contact with dogs, coinfection with *Toxoplasma*	Neurologic and psychiatric: cognitive delays; epilepsy; ocular manifestations	Pulmonary: diminished lung function asthma	Not well established	[13]–[22]
Cysticercosis	Up to 41,000–169,000 infected persons; likely widely under-recognized, with only an estimated 1,000 hospitalized cases diagnosed	Hispanic immigrants	Neurologic: epilepsy; chronic headaches	None	Not well established	[24]–[28]
Chagas Disease	300,167 cases	Hispanic-Americans	Cardiovascular: cardiomyopathy neurysms; conduction disturbances; sudden death	Gastrointestinal: megaviscera	Congenital: congenital Chagas disease syndrome	[29]–[38]
Toxoplasmosis	1.1 million new cases annually, including 21,505 cases of ocular toxoplasmosis and up to 4,000 cases of congenital toxoplasmosis	African-American ethnicity and poverty	Neurologic and psychiatric: cerebritis; schizophrenia; bipolar and other mood disorders	Ocular: retinitis and retinal scars and other ocular findings	Congenital toxoplasmosis syndrome: hydrocephalus; chorioretinitis; intracranial calcifications; cognitive deficits; hearing loss	[39]–[47]
Trichomoniasis	7.4 million new cases annually	African-American ethnicity—10 times more common	Genitourinary: vaginitis; pelvic inflammatory disease; pregnancy complications	HIV coinfections	Neonatal infections	[48]–[52]
Total	Approximately 12 million incident or prevalent infections, including some people living with chronic sequelae					

Features that produce clinical manifestations and sequelae similar to selected noncommunicable diseases.

## Neglected Helminthic Infections

Toxocariasis and cysticercosis are two of the most common parasitic worm infections. Toxocariasis occurs disproportionately in the southern US. Less is known about the distribution of cysticercosis, but it also tends to concentrate in southern and southwestern states with large Hispanic American populations [12].

### Toxocariasis

Toxocariasis is a soil-transmitted helminth infection and zoonosis that results from accidental ingestion of *Toxcara canis* or *T. cati* eggs found in soil contaminated with dog or cat feces, respectively. The resulting larval migrations through the lungs, liver, and eyes continue for months or even years to produce several different inflammatory conditions that include visceral toxocariasis, ocular toxocariasis, and covert toxocariasis with elevated serum immunoglobulin E (IgE) and eosinophilia [13]. Toxocariasis is widespread and is likely our nation's most common helminthiasis [13]. Much of what we currently know about the prevalence and risk factors for toxocariasis are from data on over 20,000 serum samples collected from the Third National Health and Nutrition Examination Survey (NHANES III) and tested by the Centers for Disease Control and Prevention (CDC) for *Toxocara* antibodies [14]. The age-adjusted seroprevalence was highest among non-Hispanic blacks (21.2%) and associated with low education, poverty, elevated lead concentrations, dog ownership, and living in the South or Northeast areas of the country [14], [15]. Toxocariasis and toxoplasmosis coinfections were also noted to be common [16]. Based on these data and the number of at-risk African Americans living in poverty, one estimate suggested that up to 2.8 million African Americans may have *Toxocara* infections [4], but there is a need to refine those estimates through further research. Among the compelling reasons to collect additional information about toxocariasis are potential links between the covert form and important pulmonary and neurologic sequelae [13]. An expanded NHANES III analysis recently linked toxocariasis to significantly diminished lung function [17] and reduced cognitive function in children [18]. Still other new studies support a link to epilepsy [19] but with conflicting evidence on the association with bronchial asthma [20]. Ocular toxocariasis is associated with vision loss, especially among children living in the South [21]. Given the high rates of toxocariasis among people living in the southern US and its association with cognitive delays, epilepsy, vision loss, and reduced pulmonary function, there is an urgent need to conduct prospective studies to confirm these links as well as to evaluate potential therapies with albendazole and other anthelminthic drugs [22]. The extent to which other soil-transmitted helminth infections still occur in the US is not known. As recently as 1982, ascariasis, trichuriasis, hookworm infection, and strongyloidiasis were prevalent in impoverished areas of the southern US and Appalachia, but over the last 30 years, there have been few if any high-quality studies to assess whether these infections still occur in historically endemic areas [23].

### Cysticercosis

Cysticercosis is a human infection with the larval form of the pork tapeworm, *Taenia solium*, which results from the accidental ingestion of eggs passed in feces from another person, typically a family member or household contact. The disease is widely distributed in Latin America as well as in Asian and African developing countries, where it is an important global cause of epilepsy and manifestations of hydrocephalus, but it has also emerged as a public health problem among Hispanic-Americans living in the US. One estimate suggests that tens of thousands of cases are present at any given time [4]. The clinical presentation of cysticercosis in Houston, Texas, is typical of that seen in other regions of the US, with neurologic manifestations (“neurocysticercosis”), especially seizures and headaches, the most common presenting signs [24]. In the patient population seen at one of Houston's two major public hospitals, more than half have parenchymal disease, which is usually a solitary cyst (often with a scolex) seen on neuroimaging [24]. However, a significant percentage of cases also show intraventricular (20%) or subarachnoid disease (12%) or calcifications (12%) [24]. In southern California, where neurocysticercosis also causes a significant health and economic burden, most of the cases require hospitalization (at which point they are most frequently diagnosed), and men were more likely to suffer from severe disease including hydrocephalus, which was more costly and required longer hospital stays [25]. The American Academy of Neurology recently issued evidence-based guidelines for the treatment of neurocysticercosis and currently recommends specific anthelminthic therapy with albendazole either with our without corticosteroids in order to decrease the number of active lesions on brain imaging studies or to reduce long-term seizure frequency [26]. Such guidelines focus on parenchymal neurocysticercosis, so additional guidelines may be required to address more complicated disease. Substance P was identified as a possible factor responsible for seizures in neurocysticercosis [27], a finding which could provide new avenues for therapy. Importantly, cysticercosis can also be acquired in the US, but the actual extent to which this occurs is unknown [25], [28]. Pilot surveillance systems in California have screened contacts of neurocysticercosis cases for tapeworm carriage, identifying a source in up to 21% of US-born cases [28]. Treatment of these tapeworm carriers can prevent disease in their contacts.

## Neglected Protozoan Infections

Three major protozoan infections have been linked to poverty in the US.

### Chagas disease

Chagas disease (American trypanosomiasis) is a chronic parasitic infection caused by *Trypanosoma cruzi*, transmitted by triatomine vectors (“kissing” bugs), and associated with severe cardiomyopathy and other life-threatening sequelae in approximately one-third of infected individuals [29]. The CDC estimates that 300,167 people live with *T. cruzi* infection in the US, including up to 45,000 with undiagnosed Chagasic cardiomyopathy [29], [30]. A new economic analysis projects the healthcare and other costs of Chagas disease in the US to be almost US$900 million annually, placing it on a similar footing with other better-known infections such as Lyme disease and methicillin-resistant *Staphylococcus aureus* infection [31]. An important gap in our knowledge of the burden of Chagas disease in the US is an accurate estimate of the number of infants being infected through mother-to-child transmission [32]. The first case of congenital Chagas disease was confirmed in 2012 [33], but it has been estimated that up to 315 infants annually (in the same order of magnitude as phenylketonuria or other inborn errors of metabolism) are born in the US with congenital Chagas disease [29]. Information on the number of infected infants and highest-risk groups could inform policies on targeted screening. Limited data suggest that up to 13% of patients with dilated cardiomyopathy in at-risk Latino populations in the US may be due to Chagas disease [34]. More accurate estimates of the burden, risk groups, and costs would inform treatment guidelines and prevent disease progression. Another major unknown is the proportion of cases in the US due to immigration from endemic areas of Latin America versus those attributable to autochthonous transmission. The triatomine vectors are widely distributed in the southern US [30], especially in Texas, where canine Chagas disease is also widespread [35]. However, to date only 23 cases of autochthonous Chagas disease have been confirmed [36]. Reasons for the lack of clarity on disease burden include limited public health surveillance and targeted surveys and poor disease-related knowledge among American physicians [37]. Few obstetricians know about the risk of congenital Chagas disease [38]. Finally, the diagnostic tests for Chagas are not easily accessible, and the antitrypanosomal drugs are highly toxic, often of limited efficacy, and contraindicated in pregnancy [30].

### Toxoplasmosis

Toxoplasmosis is a parasitic infection of humans and numerous animal species. Transmission of *Toxoplasma gondii* to humans occurs through either ingestion of cysts found in meat or oocysts found in water or soil contaminated by cat feces [39]. Based on NHANES 1999–2004 and the 2009 US census data, almost 1.1 million people are infected each year, including more than 21,505 people who develop ocular lesions [40]. While overall the prevalence of toxoplasmosis has declined from the prior decade, the disease still occurs disproportionately among non-Hispanic blacks and people living in poverty [39]. Up to 4,000 cases of congenital toxoplasmosis also occur annually [41]. Interestingly, a new test that detects antibodies to sporozoites has recently suggested that most congenital infections result from ingestion of *T. gondii* oocysts (zoonotically transmitted from cats) during pregnancy [42]. There are also data to indicate that severe disease resulting from congenital toxoplasmosis is more common in the US than in Europe [43]. However, despite this new information, there is a low level of awareness among obstetricians about toxoplasmosis and how to prevent infection [44]. The burden of *T. gondii* infection may extend beyond well-known manifestations that include ocular disease, congenital defects, and severe disease in the immunocompromised host. Some recent studies have also indicated an association between seropositivity and various psychiatric conditions, including schizophrenia, bipolar and other mood disorders, and suicide attempts [45]–[47]. Addressing important gaps in our understanding of this disease, such as estimates of incidence of congenital toxoplasmosis and cost/benefit of screening; elucidation of the association between *T. gondii* infection and mental illness; and improved diagnostic tests and treatments, would enable better prevention and control in the US.

### Trichomoniasis

Trichomoniasis is a common sexually transmitted parasitic infection with more than 7 million cases annually in the US, where it is a leading cause of vaginitis, preterm labor, and pelvic inflammatory disease [48]. Data collected over the last decade has also revealed an important link with HIV/AIDS, as women with *Trichomonas vaginalis* infection exhibit increased HIV viral shedding, which has been shown to decrease following antiparasitic chemotherapy [49]. Indeed, a significant number of HIV transmission events from HIV-infected women may be attributable to trichomoniasis coinfections [50]. The prevalence of trichomoniasis is more than ten times higher among black women than non-Hispanic white women [51]. Other factors associated with Trichomonas infection in this national sample included poverty, low educational level, increasing age, high number of sex partners, being born in the US, douching, and having a concurrent chlamydial infection [51]. Nitroimidazoles (metronidazole or tinidizole) are the only class of drug available in the US for treatment. Low levels of metronidazole resistance are now widespread among *T. vaginalis* isolates in US urban centers [52]. The CDC has also received isolates that have been resistant to tinidazole. In addition, some women are allergic to the nitroimidazoles and require desensitization, so other drugs are urgently needed. Addressing important gaps in the epidemiology of this disease, including the role of asymptomatic infections in disease transmission and the role of male infections, is needed to inform prevention policies.

### Other protozoan infections

Two other intestinal protozoan infections, i.e., cryptosporidiosis and giardiasis, are also common in the US, where they are neither linked to neglect nor poverty. Both diseases were reviewed recently with respect to their epidemiology in the US [53], [54]. Briefly, both infections are more prevalent in the northern US and exhibit their highest incidence during the summer months with links to recreational water use [53], [54]. Cyclosporiasis is a parasitic infection linked to food-borne illness, also with high incidence during the summer months [55].

## Urgent Needs and Future Directions

The neglected parasitic infections are not rare conditions in the US. Instead, they affect at least 12 million Americans, either through new infections (e.g., trichomoniasis) or from prevalent persistent infections resulting in chronic sequelae. However, these diseases typically go undiagnosed because of poor awareness among health care providers as well as the relative inaccessibility or unavailability of the diagnostic tests. Confirmatory diagnostic testing for these parasitic infections requires serologic testing that detects antibody against antigens obtained from whole parasites that are typically unavailable in most clinical laboratories. Therefore, there is an urgent need to develop improved diagnostic reagents (including recombinant antigens) and point-of-care tests. The chronic and disabling features of neglected parasitic infections can resemble selected noncommunicable diseases, which further compounds diagnostic difficulties and their lack of recognition. As examples, few health care providers might recognize toxocariasis and toxoplasmosis as underlying causes of pediatric cognitive deficits and developmental delays, toxocariasis as a cause of asthma, toxocariasis and cysticercosis as etiologies of epilepsy, or Chagas disease as a cause of heart disease. In addition to the lack of diagnostic tools, better drugs are urgently needed to either overcome resistance or have a better safety profile. There is no Food and Drug Administration (FDA)-approved drug for Chagas disease in the US, which limits the ability to scale up a treatment program for thousands of people in the US. The drugs, however, are available under investigational protocols from the CDC. Almost all of our current estimates of disease prevalence, incidence, or disease burden are based on limited testing or NHANES surveys. While blood products are screened for *T. cruzi* antibody [36], [56], such activities underestimate the true prevalence and geographic distribution of an infection like Chagas disease that occurs mostly among the poor [30]. For some diseases like Chagas disease or neurocysticercosis, for which there is a potential public health response such as screening children of infected mothers or screening household contacts of neurocysticercosis for taeniasis, public health surveillance is appropriate but seldom conducted except in a few states. Currently, Chagas disease is reportable in only four states and neurocysticercosis in five states, but even in these states there are few if any programs of active surveillance. Such limited surveillance activities hinder efforts to assess disease burdens, identify at-risk populations, and elucidate modes of transmissions. Finally, we need programs of health education and advocacy to promote awareness for the neglected parasitic infections and to shape policies for control and prevention. Pediatricians and obstetricians in particular can play a major role in advising families how to prevent these diseases. Communities need to enact and enforce regulations that prohibit pet access to children's play areas in public parks and programs to prevent *T. canis*, *T. cati*, and *T. gondii* zoonotic transmission from dog and cat feces. Directing attention and resources to the neglected parasitic infections would provide a cornerstone for a broader approach to help the most impoverished and marginalized Americans.
